# Towards Profiling of the G-Quadruplex Targeting Drugs in the Living Human Cells Using NMR Spectroscopy

**DOI:** 10.3390/ijms22116042

**Published:** 2021-06-03

**Authors:** Daniel Krafčík, Eva Ištvánková, Šimon Džatko, Pavlína Víšková, Silvie Foldynová-Trantírková, Lukáš Trantírek

**Affiliations:** 1 Central European Institute of Technology, Masaryk University, Kamenice 753/5, 625 00 Brno, Czech Republic; daniel.krafcik@ceitec.muni.cz (D.K.); eva.maturova@ceitec.muni.cz (E.I.); simon.dzatko@ceitec.muni.cz (Š.D.); pavlina.viskova@ceitec.muni.cz (P.V.); 2 National Centre for Biomolecular Research, Masaryk University, Kamenice 5, 625 00 Brno, Czech Republic; 3 Institute of Biophysics, Czech Academy of Sciences, Královopolská 135, 612 65 Brno, Czech Republic

**Keywords:** in-cell NMR, G-quadruplex, ligand, drug, Bcl2, telomeric DNA, KRAS, BRACO19, PhenDC3, NMM

## Abstract

Recently, the ^1^H-detected in-cell NMR spectroscopy has emerged as a unique tool allowing the characterization of interactions between nucleic acid-based targets and drug-like molecules in living human cells. Here, we assess the application potential of ^1^H and ^19^F-detected in-cell NMR spectroscopy to profile drugs/ligands targeting DNA G-quadruplexes, arguably the most studied class of anti-cancer drugs targeting nucleic acids. We show that the extension of the original in-cell NMR approach is not straightforward. The severe signal broadening and overlap of ^1^H in-cell NMR spectra of polymorphic G-quadruplexes and their complexes complicate their quantitative interpretation. Nevertheless, the ^1^H in-cell NMR can be used to identify drugs that, despite strong interaction in vitro, lose their ability to bind G-quadruplexes in the native environment. The in-cell NMR approach is adjusted to a recently developed 3,5-bis(trifluoromethyl)phenyl probe to monitor the intracellular interaction with ligands using ^19^F-detected in-cell NMR. The probe allows dissecting polymorphic mixture in terms of number and relative populations of individual G-quadruplex species, including ligand-bound and unbound forms in vitro and in cellulo. Despite the probe’s discussed limitations, the ^19^F-detected in-cell NMR appears to be a promising strategy to profile G-quadruplex–ligand interactions in the complex environment of living cells.

## 1. Introduction

Nucleic acids (NAs), in particular non-B DNA structures, are an important class of drug targets [1,2]. The discoveries and development of DNA-targeting drugs (ligands) heavily rely upon high-throughput screening (HTS) of the chemical libraries in vitro and in silico [3,4]. The screenings utilize a broad spectrum of biophysical or computational methods to evaluate ligands’ capacity to bind the target [4,5,6,7]. However, these methods are generally limited to non-native conditions. Unfortunately, the ligand-binding capacities determined under non-native conditions do not, in many cases, reflect the efficacy that the ligands display in vivo, where intracellular factors might drastically affect the structure of the target [8]. Significantly, alternative targets and various environmental factors can influence DNA formation and kinetic stability of NA–ligand complexes. The alternative cellular (off-)targets in cells are responsible for the vast majority of failures of the HTS positive hits in the pre-clinical phase [9].

There have been outgoing efforts to increase the success rate of HTS-based methods by involving intracellular factors in the process. The most commonly used approaches have relied on using formulated buffer solutions to recapitulate non-specific properties of the cellular environment. These factors might include: the composition mimicking the simultaneous presence of various ions (K^+^, Na^+^, Mg^2+^, Ca^2+^); synthetic additives (polyethylene glycol, Ficoll) emulating the molecular crowding and reduced dielectric permittivity of the cellular interior; micelles and nanopores simulating cellular confinements; the genomic DNA isolated from cells imitating the presence of genomic off-targets [10]. These factors notably impact DNA structure/dynamics and the capacity of small molecular weight compounds to bind them in vitro [11,12,13,14,15]. However, the quantitative in vitro structural studies and interaction data strongly depend on the subjective choice of the environmental model. The main limitation of using the formulated solution has been the absence of appropriate reference (in vivo) data.

Over the past ten years, in-cell spectroscopic methods, in particular, in-cell nuclear magnetic resonance (NMR), electron paramagnetic resonance (EPR), and Förster resonance energy transfer (FRET), have emerged as an efficient and viable alternative to the characterization of NA structures and interactions in the artificial conditions of buffers [8,16,17,18,19,20,21,22,23,24,25,26,27,28,29,30,31,32,33,34,35,36,37,38,39]. These methods allow the quantitative description under physiologically relevant conditions, i.e., in living cells. Among these methods, the in-cell NMR spectroscopy is the only one enabling high/atomic resolution readout on structure and interactions of targeted NAs. Until recently, the in-cell NMR analysis was restricted to *Xenopus laevis* (African frog) oocytes. The in-cell NMR in this cellular model has been mainly used to address NA-based drug targets’ physiological conformations [34,35,36,37,38,39,40,41]. Unfortunately, the only reported attempt to extend the technology to characterize DNA–ligand interaction failed to provide NMR spectra of sufficient quality to allow their interpretation [22]. In 2018, two distinct groups adopted the in-cell NMR approach to characterize the structure of NAs in living human cells [25,42]. The approaches have proven helpful for validating DNA target structure in vivo [30,42,43,44] and for a semi-quantitative characterization of the ligand binding to double-stranded (ds)DNA and RNA aptamers [8,33].

Here, we assess the application potential of in-cell NMR spectroscopy to profile DNA G-quadruplexes (G4s) targeted by ligands. G4s are four-stranded DNA structures formed from tetrads, consisting of guanine residues stabilized by hydrogen bonds of Hoogsteen-type [45,46,47,48]. G4s are polymorphic compared to dsDNA and heavily dependent on in vitro environmental conditions, under which they might adopt multiple, often coexisting, folding topologies [49,50,51,52,53,54,55]. The structure that a given G4 adopts under in vitro conditions is not necessarily the same as that in vivo [34,40]. The same applies to the ligand-binding potential [8]. The environmentally promoted polymorphism, specifically the lack of information on the active biological structure of G4, hampers the attempts for rational drug design and virtual screening applications. Disregarding both the polymorphism and cellular context in HTS, particularly ligand off-targeting in vivo, results in a high failure rate during the development of specific G4-binding drugs.

Compared to conventional methods applied in HTS, the use of in-cell NMR provides a possibility to characterize interactions between the target’s physiologically relevant structure and the ligand in the cellular context [8,33,56,57,58,59]. Thus far, the in-cell NMR studies of NA-ligand binding have relied on the interpretation of ^1^H-detected in-cell NMR spectra. However, the ^1^H in-cell NMR spectra of polymorphic G4s and their complexes with drug-like molecules suffer from severe signal broadening and overlap, preventing their interpretation. We show that the use of G4 constructs modified with 3,5-bis(trifluoromethyl)phenyl tag, recently developed by Xu et al. [37,38,60], combined with ^19^F-detected in-cell NMR, can help overcome the problems of ^1^H-detected in-cell NMR. Our data show ^19^F in-cell NMR can become a promising strategy to profile G4–ligand interactions in the complex environment of living cells.

## 2. Results

### 2.1. Towards Characterization of G4–Ligand Interactions In Situ Using In-Cell NMR: Initial Screening

There are several general preconditions to be fulfilled for the successful application of the in-cell NMR experiment. First, the approach presumes the intact delivery of the DNA–ligand complex into the cellular interior; the transfection step must not irreversibly compromise the integrity of the complex. Both the DNA target and DNA–ligand complex need to be non-toxic for the cells. Additionally, the preformed complex should localize into the physiologically relevant cellular compartment, a cell nucleus in the case of DNA. Finally, due to the high spectral background from cells, the 1D ^1^H in-cell NMR spectra analysis is usually limited to the imino region of the spectrum. The successful application of in-cell NMR spectroscopy to study DNA–ligand interactions presumes distinctive imino spectral fingerprints between unbound and bound DNA. All these preconditions need to be addressed before actual in-cell NMR measurements.

**Figure 1:**
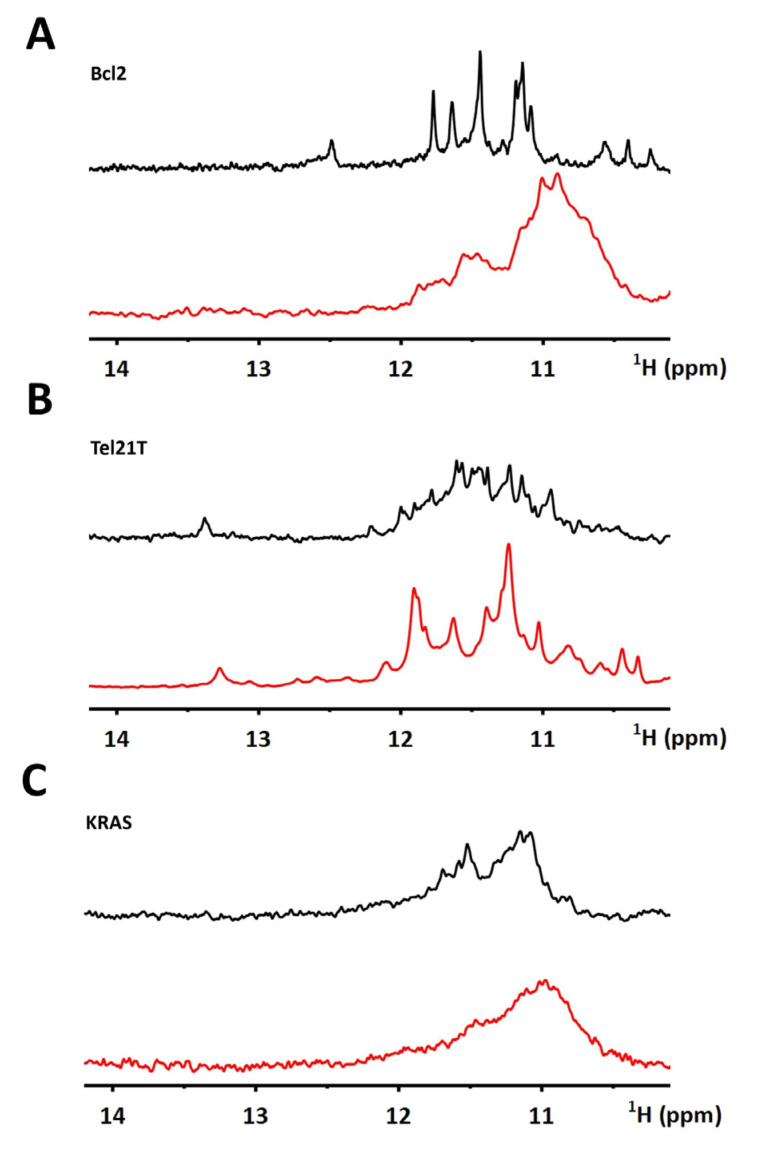
Imino regions of 1D ^1^H NMR spectra of Bcl2 (**A**), Tel21T (**B**), and KRAS (**C**), acquired in EC buffer in the absence (black) and the presence (red) of BRACO19. The spectra were measured at 20 °C. 1:2 DNA to ligand ratio was used for Bcl2, and 1:1 ratio for Tel21T and KRAS.

Figure 1 provides the representative results of the initial in vitro NMR screenings for the three most common scenarios for G4-based targets and their complexes.

*Scenario1*—*target and the corresponding complex provide well-resolved and unresolved in vitro NMR spectra, respectively*. Figure 1A (black line) shows the imino region of 1D ^1^H NMR spectrum of Bcl2 (cf. Supplementary Information Table S1), a construct from a G4 forming regulatory segment from the promoter region of the Bcl2 gene [61]. The spectrum acquired in the EC buffer shows ~12 relatively well-resolved signals in the area between 10–12 ppm. The number and positions of the signals are coherent with forming a single three-tetrad G4 topology [61]. NMR spectrum changes upon the addition of BRACO19 (Figure 1A, red line), a validated G4 binding ligand (cf. Supporting Information Figure S1) [62,63,64,65]. The change indicates the Bcl2–BRACO19 complex formation displaying multiple unresolved signals of various G4 forms. The spectrum’s complexity prevents its interpretation in terms of the target’s free and bound form population.

*Scenario 2*—*target and the corresponding complex provide unresolved and well-resolved in vitro NMR spectra, respectively*. Figure 1B (black line) shows the imino region of 1D ^1^H NMR spectrum of Tel21T (cf. Supplementary Information Table S1), a construct from a G4 forming segment from the natural variant of the human telomeric DNA [66]. The spectrum acquired in the EC buffer shows multiple unresolved signals. The number of signals notably exceeds that expected for a single G4 topology, which Tel21T adopts in K^+^ buffer (cf. Supplementary Information Figure S3). The overall spectral appearance is suggestive that in EC buffer Tel21T exists as a mixture of coexisting conformations/topologies. Similar to Scenario 1, the spectrum dramatically changes upon the addition of BRACO19 (Figure 1B, red line). In contrast to the unresolved character of the Tel21T spectrum, the Tel21T–BRACO19 complex is well-resolved and, most importantly, notably distinct from that of the free target.

*Scenario3—target and the corresponding complex provide unresolved in vitro NMR spectra*. Figure 1C shows the imino region of the 1D ^1^H NMR spectrum of KRAS (cf. Supplementary Information Table S1) [67], a construct from a G4 forming regulatory segment from the KRAS gene promoter and that of the KRAS–BRACO19 complex, respectively. While the spectra show differences, their unresolved character precludes their interpretation, even under simplistic in vitro conditions.

Overall, our data show that G-quadruplex targets and their complexes falling into Scenario 3 are not generally suited for subsequent in-cell NMR analysis compared to the situations represented by Scenario 1 and 2.

**Figure 2:**
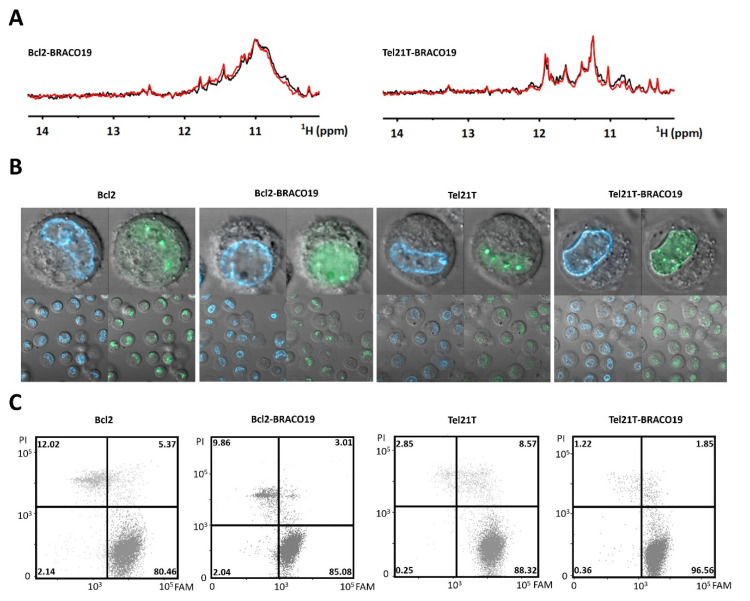
(**A**) Imino regions of 1D ^1^H NMR spectra of Bcl2 (left) and Tel21T (right) acquired before (black line) and after (red line) “mock” electroporation in EC buffer. (**B**) Confocal microscopy images taken with 63 ×/1.2 C-Apochromat objective of RPE cells transfected with Bcl2, Bcl2–BRACO19, Tel21T, and Tel21T–BRACO19. The green color indicates the localization of (FAM)-DNA/(FAM)-DNA–ligand complex. The blue color corresponds to a cell nucleus stained by Hoechst 33342. (**C**) Double staining (PI/FAM) FCM analysis of transfected RPE cells with the DNA and DNA–ligand complexes. Percentages (the numbers in the FCM plots) of viable non-transfected cells, viable DNA/DNA–ligand containing cells, non-transfected dead/compromised cells, and transfected dead/compromised cells containing DNA/DNA–ligand are indicated in left-bottom, right-bottom, left-top, and right-top quadrants, respectively.

The targets, once passed through preliminary in vitro NMR screening, were subjected to further tests prior to in-cell NMR experiment [8,28]. To assess the impact of transfection procedure, the NMR spectra of the complex acquired before and after the electroporation step were compared in the absence of cells. Figure 2A shows 1D ^1^H NMR spectra of the Bcl2–BRACO19 and Tel21T–BRACO19 complexes measured in EC buffer before and after the “mock” electroporation. For both complexes, the spectra before and after the mock electroporation were essentially identical. This experiment confirmed that the electroporation does not compromise the integrity of either the complexes or the DNAs.

Figure 2B shows confocal microscopy images of cells transfected with the fluorescein-modified (FAM) G-quadruplex targets. Both the Bcl2 and Tel21T constructs showed physiologically relevant nuclear localization with or without added ligands. Flow cytometry plots acquired on the DNA transfected cells confirmed neither introduced DNA nor did its complex impact cells’ viability that exceeded 80% (Figure 2C). Both Bcl2 and Tel21T in complex with BRACO19 appear suitable targets for evaluating the complex stability in cells.

### 2.2. ^1^H-Detected NMR as a Coarse-Grained Tool for Profiling the G4–Ligand Interactions in Living Cells

**Figure 3:**
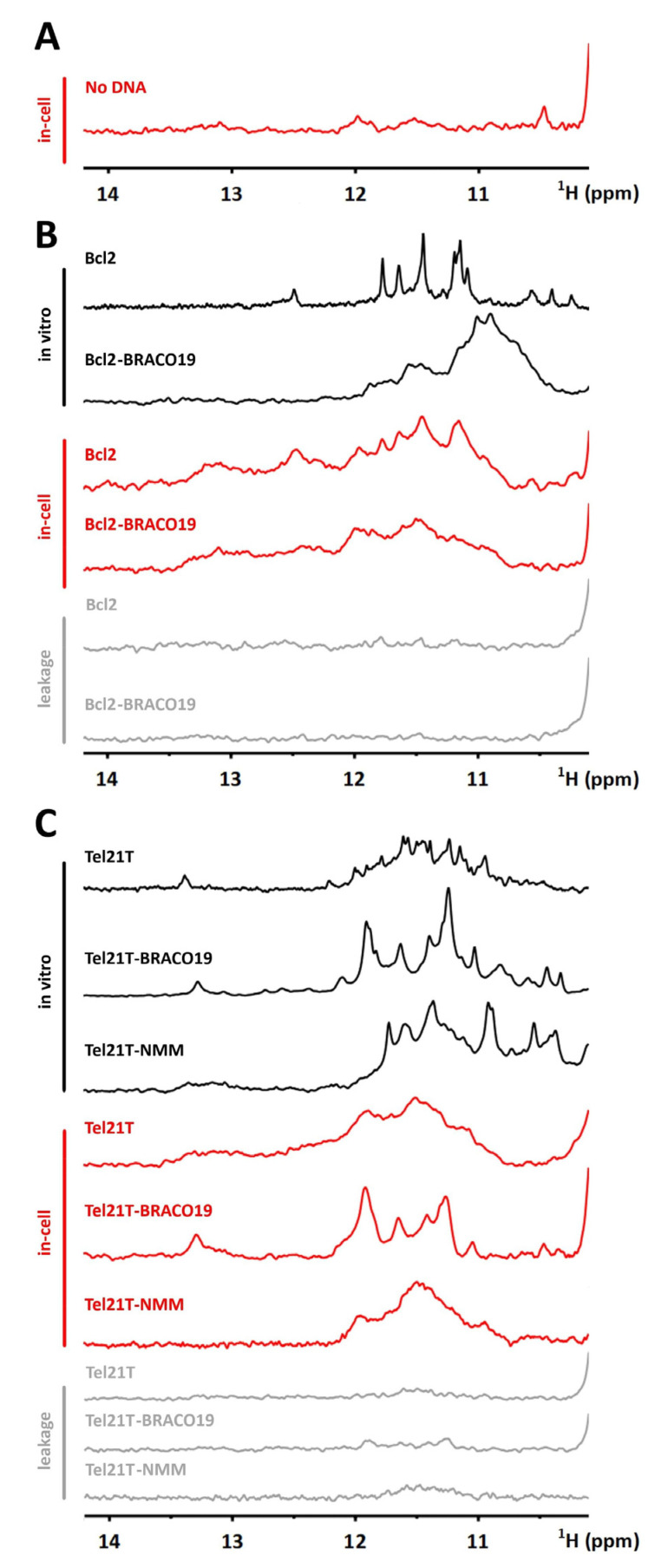
(**A**) Imino region of 1D ^1^H NMR spectra of “empty” RPE cells. (**B**) Imino regions of 1D ^1^H NMR spectra of Bcl2 and Bcl2–BRACO19 complex acquired in EC buffer (black line) and cells (red line). (**C**) Imino regions of 1D ^1^H NMR spectra of Tel21T, Tel21T–BRACO19, and Tel21T–NMM complex acquired in EC buffer (black line) and cells (red line). NMR spectra presented in gray in (**B**,**C**) correspond to the respective in-cell NMR leakage controls.

Bcl2 and Tel21T were transfected into RPE cells at the scale required for in-cell NMR measurements (cf. Material and methods). In parallel, the suspension of RPE cells was subjected to electroporation in the absence of DNA and ligand. While the imino regions of 1D ^1^H in-cell NMR spectra of Bcl2 and Tel21T were to serve as spectral fingerprints of an unbound form of DNA, the corresponding region of “empty” RPE cells was to provide information on the contribution of the cellular background to the spectra of the targets (Figure 3A).

Following the in-cell NMR spectrum acquisition, the medium surrounding the transfected cells was collected. The medium NMR spectrum, so-called leakage control, reports on the target/target–drug complex leakage from cells during in-cell NMR data acquisition.

In contrast to the in vitro NMR spectrum of Bcl2, the signals in the corresponding in-cell NMR spectrum were considerably broadened (Figure 3B). Nonetheless, the similarity in line-shapes between in vitro and in-cell NMR spectra suggests that Bcl2 adopts the same conformation in vitro as in cells. However, the in-cell NMR spectrum of the Bcl2–BRACO19 complex matched neither the reference spectrum of the unbound nor the target’s bound form, and its compromised quality did not allow spectral deconvolution. In this case, one should avoid further interpretation of the in-cell NMR data.

Similar to the Bcl2 case, the in-cell NMR spectrum of ligand-free Tel21T was unresolved, and signals significantly broadened (Figure 3C). In this case, the spectrum’s interpretation relies only on the composite signal envelope’s shape and position, which precludes unambiguous identification of G4 topology. In contrast, the spectrum of the Tel21T–BRACO19 complex is well-resolved, and its overall pattern corresponds to the in vitro NMR spectrum. The matching spectral fingerprints [35] suggest that Tel21T and BRACO19 form a stable complex in the crowded intracellular space.

The example of the Tel21T–BRACO19 complex is representative of the positive ligand validation; the BRACO19 binds the target (Tel21T) under both in vitro and intracellular conditions. The case of the Tel21T in the complex with NMM (cf. Supplementary Information Figure S1), another in vitro validated G4 binding ligand [68,69], illustrates the negative outcome of in-cell NMR-based ligand validation. The in vitro NMR spectrum of the Tel21T–NMM complex is relatively well-resolved and notably distinct from that of the Tel21T (target) alone (Figure 3C), providing another example of Scenario 2. Similar to the Tel21T–BRACO19, the transfection of the Tel21T–NMM complex in RPE cells does not compromise cells’ viability (Supplementary Information Figure S2). The transfected complex localizes in the cell nucleus (Supplementary Information Figure S2). However, in contrast to the Tel21T–BRACO19 in-cell NMR spectrum, the in-cell NMR spectrum of Tel21T–NMM shows no similarity to the in vitro spectrum of the complex (Figure 3C). At the same time, the shape of the envelope closely resembles the spectrum of the unbound Tel21T. Altogether, the (in-cell) NMR data indicate that the Tel21T–NMM complex is unstable in the cellular context.

### 2.3. Potential of ^19^F-Detected NMR for Monitoring G4–Ligand Interactions in the Intracellular Space

**Figure 4:**
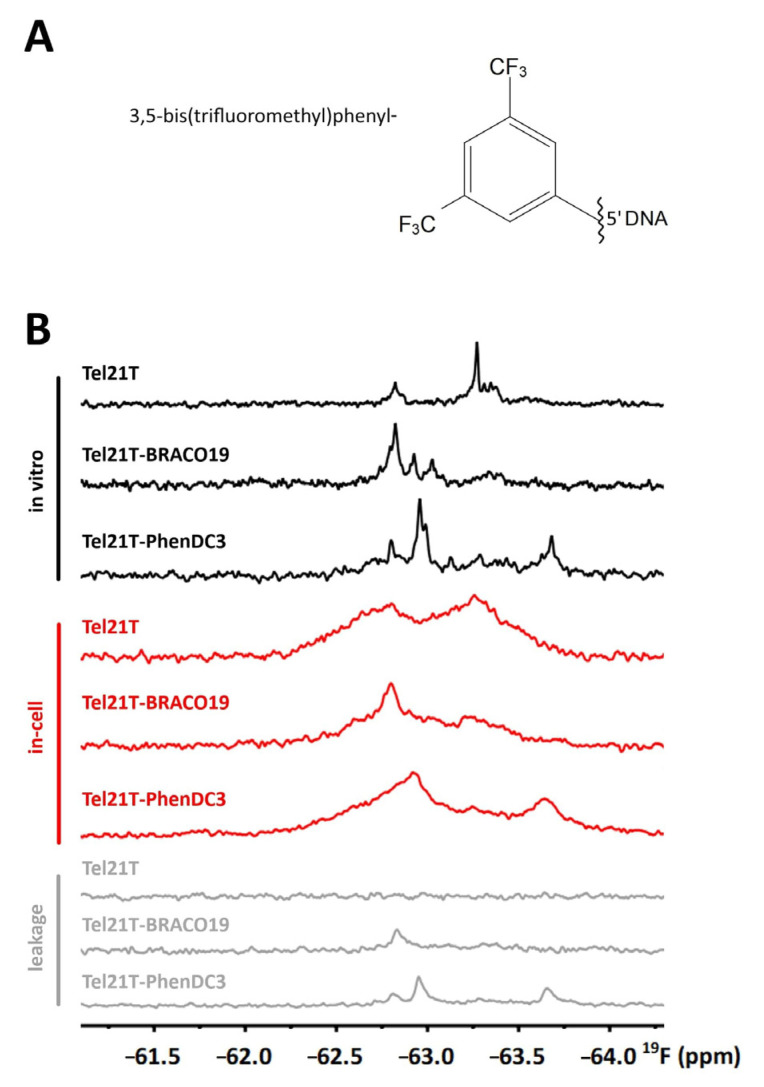
(**A**) The scheme of the ^19^F-probe attached to the DNA—cf. [37]. (**B**) 1D ^19^F NMR spectra of F-Tel21T, F-Tel21T–BRACO19, and F-Tel21T–PhenDC3 acquired in EC buffer (black line) and cells (red line). NMR spectra presented in gray correspond to the respective in-cell NMR leakage controls.

Our data suggested that the signal overlap in the imino region was one of the main limitations in applying the ^1^H-detected in-cell NMR spectroscopy to profile G4-binding ligands. As recently demonstrated by Xu et al. [30,37,43], using ^19^F-detected in-cell NMR can effectively evade both the cellular background’s interference and the signal overlap in the ^1^H NMR spectra. ^19^F is not naturally present in the intracellular space; the ^19^F-detected in-cell NMR spectra are free of spectral background. Xu et al. showed that the 3,5-bis(trifluoromethyl)phenyl group (Figure 4A), referred to as ^19^F-probe onwards, linked to 5’-terminus of DNA allows discrimination of G4 topologies using ^19^F-detected in-cell NMR [37,70].

Here, we examined the ^19^F-probe to monitor G4–ligand interactions in the intracellular space. Figure 4B shows 1D ^19^F in vitro/in-cell NMR spectra of Tel21T modified with ^19^F-probe (F-Tel21T) in the absence and the presence of BRACO19 and an additional validated G4-binding ligand, PhenDC3 (cf. Supplementary Information Figure S1) [71,72,73,74,75]. 1D ^19^F in vitro NMR spectrum of the F-Tel21T displays two dominant signals located at about –62.80 and −63.25 ppm (Figure 4B). The intensity of the signal at −62.80 ppm is ~30% of that at −63.25 ppm, suggesting that F-Tel21T forms two major coexisting conformations under in vitro conditions with populations roughly estimated to be 1:3. The 1D ^19^F in-cell NMR spectrum of F-Tel21T shows two broad signals at essentially identical positions as those in the in vitro spectrum (Figure 4B). The similar signal positions indicate that F-Tel21T adopts identical conformations in EC buffer and in cells. However, the 1:1 signal ratio in the in-cell NMR spectrum compared to the 1:3 signal ratio in the in vitro NMR spectrum suggests cell environment-induced changes in the populations of the conformational states.

The F-Tel21T in vitro ^19^F NMR spectrum significantly differs from F-Tel21T in vitro ^19^F NMR spectra acquired in the presence of the ligands (BRACO19 and PhenDC3)—cf. Figure 4B. The F-Tel21T–BRACO19 spectrum features three signals; a strong signal located at −62.80 ppm and two small signals positioned at −62.90 and −63.05 ppm (Figure 4B). The strongest signal position in the F-Tel21T–BRACO19 spectrum matches with the position of the minor signal in the spectrum of F-Tel21T, which suggests preferential binding of BRACO19 to the corresponding F-Tel21T conformation. Two additional (small) signals in the spectrum of F-Tel21T–BRACO19 have no equivalent in the F-Tel21T spectrum (Figure 4B). The interpretation of these signals is ambiguous. They might correspond to other ligand-induced F-Tel21T structures or arise due to the ligand’s direct interaction with the ^19^F probe (see below). Notably, the in vitro ^19^F NMR spectrum pattern of F-Tel21T–BRACO19 differs from that of F-Tel21T–PhenDC3 (Figure 4B). The spectrum of F-Tel21T–PhenDC3 shows two dominant peaks located at about −62.95 and −63.70 ppm, suggesting the co-existence of at least two distinct PhenDC3-induced structures of F-Tel21T. Additionally, the spectrum displays three minor signals. Two out of three minor signals correspond to initial (unbound) F-Tel21T conformations.

Most importantly, in-cell 1D ^19^F NMR spectra of F-Tel21T–BRACO19 and F-Tel21T–PhenDC3 recapitulate the appearance of the corresponding in vitro NMR spectra. The similarity between the in vitro and in-cell NMR spectra of F-Tel21T–BRACO19 and F-Tel21T–PhenDC3 indicates that corresponding complexes are stable in the intracellular space.

### 2.4. Limiting Factors for Using 3,5-Bis(trifluoromethyl)phenyl Group as a Probe for Monitoring G4–Ligand Interactions in Cells

**Figure 5:**
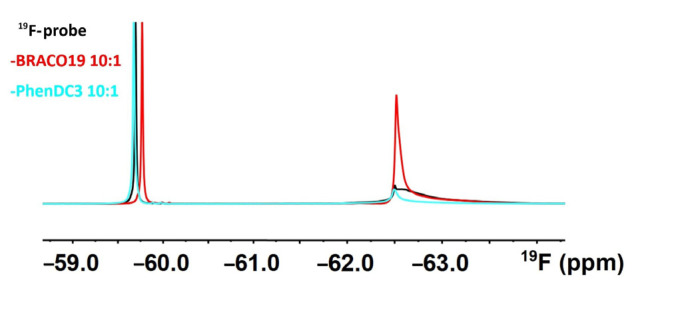
1D ^19^F NMR spectra of the ^19^F-probe (cf. Figure 4A) in the absence (black) and the presence of BRACO19 (red) and PhenDC3 (blue).

The application of the ^19^F-probe to monitor G4–ligand interactions implicitly presumes that the probe reports exclusively on the structure of the target. In other words, it presumes that the probe is not interacting with the ligand. Therefore, we acquired the 1D ^19^F in vitro NMR spectra of the ^19^F-probe (detached from the DNA) in the ligand (BRACO19, PhenDC3) absence and presence. As shown in Figure 5, ligand BRACO19 changed the appearance of the ^19^F NMR spectrum of the free tag, suggesting a possible interaction. Adding BRACO19 leads to both ~0.1 ppm shift of the probe signal and a new signal’s appearance at ~−62.5 ppm, implying (likely stacking) interaction of BRACO19 with the probe’s aromatic ring. The interactions between the probe and a ligand might, to some extent, bias in-cell NMR spectra interpretation and contribute to the ^19^F signal broadening in the in-cell NMR spectra.

The use of the 3,5-bis(trifluoromethyl)phenyl group as a ^19^F-probe increases NMR detection sensitivity due to the six isochronous fluorine atoms. While the increased sensitivity is advantageous under in vitro conditions, it complicates the in-cell NMR data interpretation. The high sensitivity of the ^19^F-probe can reveal even low levels of the leaked target (Figure 4, gray line). Detected leakage causes issues in interpreting the in-cell NMR spectra (as discussed in detail below).

## 3. Discussion

We probed the possibility of using both ^1^H- and ^19^F-detected in-cell NMR spectroscopy to monitor G4–ligand interactions in living mammalian cells. We showed that RPE cells could be transfected with G4 DNA and preformed G4–ligand complexes at concentrations required for their detection by in-cell NMR experiment. Neither G4 DNA nor G4–ligand complexes were affected by an electroporation pulse applied upon transfection. The transfected DNA oligonucleotides with and without added ligand were mainly localized in the cell nucleus and did not compromise the viability of the cells. Our data imply that a combination of RPE cells and electroporation as the transfection method appears more suitable for G4 in-cell NMR sample preparation than the previously used approach based on HeLa cells and pore-forming toxins. First, it does not compromise the viability of the cells. The cells transfected using the pore-forming toxin strategy have viability typically between 50–60% [25,37,38].In contrast, the viability of cells transfected via electroporation usually ranges between 80–95%. Second, it limits the leakage of transfected DNA from cells throughout NMR spectra acquisition. The leakage of transfected exogenous NA almost always contaminates ^1^H-detected NMR spectra of cells transfected with the toxin-based approach [25,37,38]. However, it essentially does not contribute to the ^1^H-detected in-cell NMR spectra of the DNA electroporated cells (cf. Figure 3).

In the Tel21T–BRACO19 and Tel21T–NMM cases, we demonstrated that the ^1^H-detected in-cell NMR-based assessment of the complex’s stability between polymorphic G4-based target and its ligand is, in principle, feasible. Our data on Bcl2 and KRAS identify the signal crowding and the lack of resolution as limiting factors in using ^1^H-detected in-cell NMR to monitor G4–ligand interactions. The crowding and unresolved character of ^1^H in-cell NMR spectra prevent their interpretation.

As demonstrated here, the use of the recently introduced 3,5-bis(trifluoromethyl)phenyl probe might, to a great extent, help overcome the limitation of ^1^H-detected in-cell NMR. The probe allows dissecting polymorphic mixture in terms of number and relative populations of individual G4 species, including bound and unbound forms in vitro and in cells. Nevertheless, our data drew attention to the probe’s limitations. The observed severe signal broadening might prohibit detection of low populated species or species not sufficiently separated by ^19^F chemical shift. The slower molecular tumbling rate due to the intracellular environment’s increased viscosity cannot fully explain the observed broadening of ^19^F (and ^1^H) signals in G4 in-cell NMR spectra (cf. Figure 3 and Figure 4). G4 interactions with proteins likely contribute to the broadening of ^1^H/^19^F signals (cf. Supporting Figure S4) and are, at least partially, responsible for the unresolved nature of the in-cell NMR spectra.

Additionally, we show that the probe might interact with some of the G4-binding ligands. The potential interaction might bias the in-cell NMR spectra interpretation. Finally, the fluorine probe’s high sensitivity reveals even a minimal amount of an extracellular, leaked target. In the target leakage case, the in-cell NMR spectra need to be regarded as a superposition of genuine in-cell and contaminating in vitro NMR spectrum. In principle, subtraction of apparent in-cell and in vitro “leakage” NMR spectra reconstitutes the genuine in-cell NMR spectrum. On a practical note, one wants to avoid spectrum reconstitution. The procedure of leakage sample collection and subjective choice of its subtraction might impact the interpretation of the reconstituted in-cell NMR spectrum. However, the adaptation of the bioreactors, established in the protein in-cell NMR field [59,76,77,78,79], allowing continuous removal of cell metabolism byproducts and leaked DNA/DNA–ligand complex during in-cell NMR data collection or discontinuous medium renewal [28,42], might provide an alternative to the spectra subtraction in the future.

## 4. Materials and Methods

**DNA oligonucleotides.** The DNA oligonucleotides (cf. Supplementary Information Table S1) and their FAM 5-terminally labeled analogs were purchased from Sigma-Aldrich (Saint-Louis, MO, USA). 5′-terminally ^19^F-modified oligonucleotide (F-Tel21T) was prepared according to a modified protocol by Bao and Xu [37,38,60].

**G-quadruplex Ligands.** BRACO19, PhenDC3, and NMM were purchased from Sigma-Aldrich (Saint-Louis, MO, USA).

**(in-cell) NMR spectroscopy.** The oligonucleotide stocks used for both in vitro NMR measurements and in-cell NMR samples were prepared as described in Viskova et al. [28]. (in-cell) NMR spectra were measured at 600 MHz or 850 MHz using a Bruker Avance Neo spectrometer (Billerica, MA, USA) equipped with a quadruple-resonance inverse cryogenic probe and triple-resonance (^1^H/^19^F-^13^C-^15^N) inverse cryoprobe, respectively. The DNA concentration of the in vitro samples in the EC buffer (140 mM sodium phosphate, 5 mM KCl, 10 mM MgCl_2_, pH = 7.2) was 100 µM unless indicated otherwise. In vitro spectra were acquired with 256 scans, in-cell 1D ^1^H-NMR, and 1D ^19^F-NMR with 1024 and 512 scans, respectively. The 1D ^1^H-NMR spectra were acquired using a JR-echo (1-1 echo) pulse sequence [80] with the zero excitation set to the water resonance and the excitation maximum set to 12 ppm. The processing of the spectra was done by the exponential apodization function and the line-broadening 5 Hz. After in-cell NMR spectrum acquisition, the 1D ^1^H-NMR spectrum of the in-cell supernatant was taken out of the NMR tube and measured using the same NMR setup. MNova v12 software (Mestrelab Research, Santiago de Compostela, Spain) was used to process (in-cell) NMR data.

**Flow cytometry and Confocal microscopy.** The control samples for the in-cell NMR experiment were prepared as described in Viskova et al. [28].

## 5. Conclusions and Perspectives

Our data demonstrate that the in-cell NMR concept can be extended to the assessment of medicinally-important G-quadruplex-based drug targets. The use of structure and interaction-sensitive ^19^F-probes might facilitate the in-cell NMR studies and holds a grand promise for DNA-drug screening using in-cell NMR in the future. However, the approach remains limited to assessing the preformed complexes’ stability in cells. Development of novel strategies allowing monitoring of the DNA–ligand complexes’ de novo formation and the next generation of ^19^F-probes are two significant future challenges.

## Data Availability

The data presented in this study are available in article and Supplementary Materials.

## Supplementary Materials

The Supplementary Materials are available online at https://www.mdpi.com/article/10.3390/ijms22116042/s1.
