# Supplementary material for: Towards Profiling of the G-Quadruplex Targeting Drugs in the Living Human Cells Using NMR Spectroscopy
Source: Int J Mol Sci. 2021 Jun 3;22(11):6042. doi: 10.3390/ijms22116042 (PMC8199861; doi:10.3390/ijms22116042)
Supplement: Supplementary file 1 [file ijms-22-06042-s001.zip › ijms-1225667-supplementary.pdf]

Supporting Information

Towards Profiling of the G-Quadruplex Targeting Drugs in the Living Human Cells Using NMR Spectroscopy

Daniel Krafčík [1,2], Eva Ištvánková [1,2], Šimon Džatko [1,2], Pavlína Víšková [1,2], Silvie Foldynová-Trantírková [3,*] and Lukáš Trantírek [1,*]

[1] Central European Institute of Technology, Masaryk University, Kamenice 753/5, 625 00 Brno, Czech Republic; daniel.krafcik@ceitec.muni.cz (D.K.); eva.maturova@ceitec.muni.cz (E.I.); simon.dzatko@ceitec.muni.cz (Š.D.); pavlina.viskova@ceitec.muni.cz (P.V.)

[2] National Centre for Biomolecular Research, Masaryk University, Kamenice 5, 625 00 Brno, Czech Republic

[3] Institute of Biophysics, Czech Academy of Sciences, Královopolská 135, 612 65 Brno, Czech Republic

* Correspondence: silvie.trantirkova@ceitec.muni.cz (S.F.-T.); lukas.trantirek@ceitec.muni.cz (L.T.)

Table S1. List of DNA oligonucleotides used.

Name	Sequence
Tel21T	GGGTTA GGGTTA GGGTTT GGG
Bcl2	GGGCAG GAGGGC TCTGGG TGGGT
KRAS	AGGGCG GTGTGG GAATAG GGAA

BRACO19

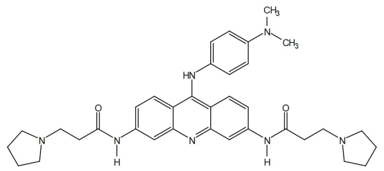

PhenDC3

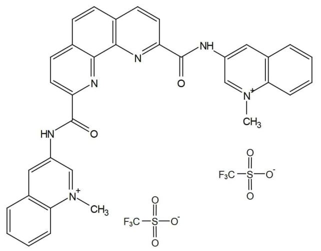

NMM

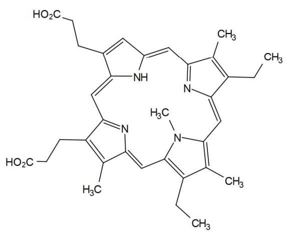

Figure S1. G-quadruplex-binding ligands used in the study.

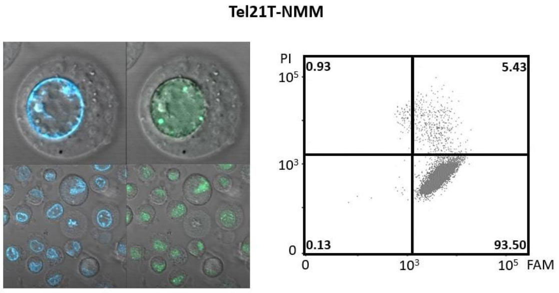

Figure S2. (Left) Confocal microscopy images of RPE cells transfected with Tel21T-NMM. The green color indicates the localization of (FAM)-DNA/(FAM)-DNA-ligand complex. The blue color corresponds to a cell nucleus stained by Hoechst 33342. (Right) Double staining (PI/FAM) FCM analysis of transfected RPE cells with the Tel21T-NMM complex. Percentages (the numbers in the FCM plots) of viable non-transfected cells, viable DNA/DNA-ligand containing cells, non-transfected dead/compromised cells, and transfected dead/compromised cells containing DNA/DNA-ligand are indicated in left-bottom, right-bottom, left-top, and right-top quadrants, respectively.

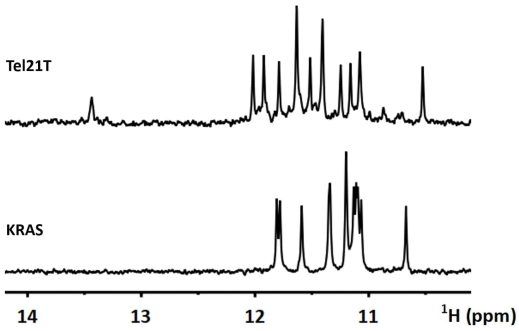

Figure S3. Imino regions of 1D ^{1}H NMR spectra of Tel21T and KRAS acquired in K^+ buffer (70 mM KCl, 20 mM KPO_i, pH = 7.0).

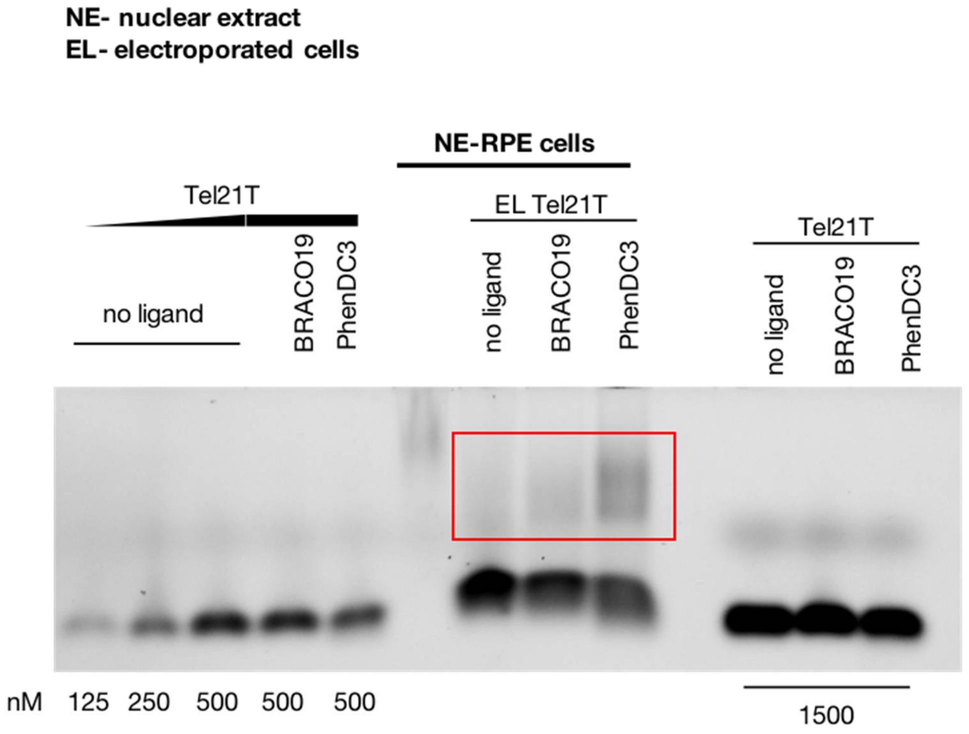

Figure S4. FAM-Tel21T -/+ ligands in the nuclear extracts from electroporated RPE cells separated on 0.8% agarose gel/1x TBE. Red box highlights putative complexes between Tel21T and nuclear proteins.
